# Supplementary material for: Distribution of high-dimensional entanglement via an intra-city free-space link
Source: Nat Commun. 2017 Jul 24;8:15971. doi: 10.1038/ncomms15971 (PMC5527279; doi:10.1038/ncomms15971)
Supplement: Supplementary Information [file ncomms15971-s1.pdf]

SI GUIDE

Title of file for HTML: Supplementary Information
Description: Supplementary Figures, Supplementary Methods and Supplementary References.

Title of file for HTML: Peer Review File
Description:

Supplementary Discussion

In the main manuscript we report on the distribution of polarization/energy-time hyperentangled photons via a free-space link. In order to assess the integrity of the atmospheric quantum communication channel we performed successive correlation measurements in superposition bases. We observed two-photon polarization interference visibility of $V_{\text{pol}} \sim 98.5\%$ and $V_{\text{e-t}} \sim 95.6\%$ for Franson interference, respectively.

While high-visibility interference in both degrees of freedom is a clear sign for the presence of hyperentanglement, these measurements do not yet quantify entanglement in the two subspaces, nor do they quantify the dimensionality of entanglement in the combined state space. Ideally, Alice and Bob would perform a complete tomography of the hyperentangled state. However, as we will show in the following, a complete state tomography is not required to obtain lower bounds on several quantitative measures of entanglement. Moreover, these lower bounds can be derived from independent visibility measurements in the energy-time and polarization subspaces, as shown in the final section of this theory supplement.

The supplementary discussion is structured as follows: First, we review the experimental setup used to measure two-photon visibilities in the energy-time and polarization degrees of freedom. Then, we show how the independently observed visibilities can be used to determine lower bounds on the concurrence and entanglement of formation in the polarization and energy-time subspaces. We conclude with a discussion of the lower bound these measurements impose on the dimensionality of entanglement in the combined state space of the entire system.

Revision of Experimental Setup

In this section we provide additional information on the experimental setup and methods used to quantify entanglement. We start by reviewing the nature of the hyperentangled target state, experimental density matrices, and visibility measurements in a notation that is more in line with the standard quantum information formalism.

Hyperentangled target state First, let us consider the ideal polarization/energy-time hyperentangled state: A strong cw pump laser with a coherence time t_{p} pumps a single nonlinear crystal inside a polarization Sagnac interferometer and produces polarization-entangled photon pairs with a coherence time $t_{\text{c}} \lesssim 1\text{ps}$. The coherence time of the pump laser is significantly longer than the coherence time of the signal and idler photons $t_{\text{c}} \ll t_{\text{p}}$, resulting in a maximally hyper-entangled state:

$$|\Psi\rangle_{\text{total}} = |\Phi\rangle_{\text{pol}} \otimes |\Phi\rangle_{\text{e-t}} = \frac{1}{\sqrt{2}} \left(|\text{H}\rangle_{\text{A}}|\text{H}\rangle_{\text{B}} + |\text{V}\rangle_{\text{A}}|\text{V}\rangle_{\text{B}}\right) \otimes \int |\tau\rangle_{\text{A}}|\tau\rangle_{\text{B}} \text{d}\tau \tag{1}$$

where H and V represent horizontally and vertically polarized photon states, τ denotes the photon emission time, and the subscripts A and B label the respective single-mode fiber for Alice and Bob. For the sake of brevity we have assumed a cw pump laser with infinite coherence time and perfectly correlated

photon pair emissions[1]. In the following discussion it will be sufficient to group pair emissions within the range $\tau = t_i - \delta t/2 \rightarrow \tau = t_i + \delta t/2$ into time bins $|t_i\rangle$ (see Fig.1). The size of these time bins is chosen to be larger than the coherence time of the SPDC photons, but smaller than the coherence time of the pump laser ($t_c \leq \delta t \ll t_p$). Hence, the energy-time state can be considered as a coherent superposition of $N \sim t_p/\delta t$ orthogonal time bins, thus constituting an N-dimensionally entangled state in the energy-time domain:

$$|\Phi\rangle_{\text{e-t}} = \frac{1}{\sqrt{N}} \sum_{i=1}^{N} |t_i\rangle_A |t_i\rangle_B \tag{2}$$

For large N it is generally not experimentally feasible to exploit the entire state space and in our proof of concept demonstration we focused on a two-dimensional subspace spanned by time-delayed states $|t\rangle$ and $|t+\tau\rangle$:

$$|\Phi\rangle_{\text{e-t}} = \frac{1}{\sqrt{2}} \left(|t\rangle_A |t\rangle_B + |t+\tau\rangle_A |t+\tau\rangle_B \right) \tag{3}$$

The total state space accessed in our experiment thus comprises the 2-dimensional polarization space and an effectively 2-dimensional energy-time subspace. More details on the definition of this state space will be provided in the following. Omitting phase dependencies the hyperentangled state of the total system can be expressed as a maximally-entangled Bell state in four dimensions:

$$|\Psi\rangle_{\text{total}} = \frac{1}{2} \left(|0\rangle_A|0\rangle_B + |1\rangle_A|1\rangle_B + |2\rangle_A|2\rangle_B + |3\rangle_A|3\rangle_B \right) \tag{4}$$

where $|0\rangle = |\text{H}, t\rangle$, $|1\rangle = |\text{H}, t+\tau\rangle$, $|2\rangle = |\text{V}, t\rangle$, $|3\rangle = |\text{V}, t+\tau\rangle$.

Polarization interference visibility and characteristic density matrix elements

Our experimental implementation (see Fig. 2a.) allowed for measurements of the following observables:

	Observable	Eigenstates
computational basis Alice	σ_A^z	$\|\text{H/V}\rangle$
computational basis Bob	σ_B^z	$\|\text{H/V}\rangle$
superposition basis Alice	$\sigma_A^\phi = \cos(\phi)\sigma_A^x + \sin(\phi)\sigma_A^y$	$\| \pm \phi\rangle = \frac{1}{\sqrt{2}} \left(\|\text{H}\rangle \pm e^{i\phi}\|\text{V}\rangle \right)$
superposition basis Bob	σ_B^x	$\| \pm 45^\circ\rangle = \frac{1}{\sqrt{2}} \left(\|\text{H}\rangle \pm \|\text{V}\rangle \right)$

where $\sigma_{A/B}^i$ denote the Pauli operators for Alice and Bob, respectively[2]. A full tomographic recon-

[1]For a more rigorous discussion of the energy-time state in SPDC, see e.g. Ref. [1]

[2]Note that the linear ±45° basis is included as the special case $\sigma_A^{\phi=0} = \sigma_A^x$.

struction of the polarization density matrix:

$$\rho_{\text{pol}} = \begin{matrix} & \text{H}_\text{A}\text{H}_\text{B} & \text{H}_\text{A}\text{V}_\text{B} & \text{V}_\text{A}\text{H}_\text{B} & \text{V}_\text{A}\text{V}_\text{B} \\ \begin{matrix}\text{H}_\text{A}\text{H}_\text{B}\\ \text{H}_\text{A}\text{V}_\text{B}\\ \text{V}_\text{A}\text{H}_\text{B}\\ \text{V}_\text{A}\text{V}_\text{B}\end{matrix} & \multicolumn{4}{c}{\begin{pmatrix} \rho_{0000} & \rho_{0001} & \rho_{0010} & \rho_{0011} \\ \ddots & \rho_{0101} & \rho_{0110} & \rho_{0111} \\ \ddots & \ddots & \rho_{1010} & \rho_{1011} \\ c.c. & \ddots & \ddots & \rho_{1111} \end{pmatrix}} \end{matrix} \tag{5}$$

is not possible from these measurements. It is, however, possible to infer lower bounds on the absolute value of various density matrix elements. In order to see this, we now consider how the measured visibilities relate to elements of the density matrix. For the visibility of joint measurements in the computational basis we have:

$$V_{\text{pol}}^{\text{H/V}} = \langle \sigma_{\text{A}}^{\text{z}} \otimes \sigma_{\text{B}}^{\text{z}} \rangle = \rho_{0000} - \rho_{0101} - \rho_{1010} + \rho_{1111} \tag{6}$$

Similarly, for the visibility in the coherent superposition basis, which is mutually unbiased to the computational basis, we have:

$$V_{\text{pol}}^{\phi} = \max_{\phi}[\langle \sigma_{\text{A}}^{\phi} \otimes \sigma_{\text{B}}^{\text{x}} \rangle] = 2\max_{\phi}[\text{Re}(\rho_{0011}e^{i\phi} + \rho_{0110}e^{i\phi})] \tag{7}$$

Energy-time interference visibility and characteristic density matrix elements Next, let us discuss the correlation measurements that were performed in the energy-time subspace and relate the Franson interference visibility to the magnitude of certain matrix elements of the energy-time density matrix. For the sake of brevity let us assume that the state of the total system $\rho_{\text{pol,e-t}}$ is a product of energy-time and polarization states:

$$\rho_{\text{pol,e-t}} = |\Phi_{\text{pol}}^{+}\rangle\langle\Phi_{\text{pol}}^{+}| \otimes \rho_{\text{e-t}} \tag{8}$$

This assumption will be justified a posteriori when we demonstrate that the experimental polarization state has a high degree of overlap with the maximally entangled Bell state, as this also implies that the polarization degree of freedom (DOF) cannot exhibit significant correlations with the energy-time space.

As discussed in the main manuscript, we employed a variant of the original Franson scheme [2,3] with unbalanced polarization interferometers to assess energy-time interference in a 2-dimensional subspace. These unbalanced polarization interferometers at Alice and Bob were implemented with 3-mm-long calcite crystals, which could be inserted before the polarization analyzers (see Fig. 2b.). The calcite crystals introduce a polarization-dependent time shift τ. In the following we omit additional phase shifts due to the

propagation through the crystal, as they can be absorbed into the phase plate in Alice's detection module. The operator describing the transformation in the polarization and energy-time space can thus be written as:

$$\mathcal{T} = |\mathrm{H}\rangle\langle\mathrm{H}| \otimes \hat{\tau} + |\mathrm{V}\rangle\langle\mathrm{V}| \otimes 1 \tag{9}$$

where 1 is the identity operator and $\hat{\tau}$ transforms the energy-time basis states (Eq. 2) as:

$$\hat{\tau}|t_i\rangle = |t_{i+1}\rangle \tag{10}$$

Note that the particular choice of delay τ introduced in the unbalanced polarization interferometer restricts our considerations to a two-dimensional subspace of the intrinsically continuous-variable energy-time space, which could be fully exploited, e.g., in experimental implementations with a variable delay line [4,5]. Since all the measurement results obtained in our experiment can be related to this 2-dimensional subspace, we define the effective energy-time density matrix as:

$$\rho'_{\text{e-t}} = \begin{pmatrix} \sum_i\langle t_it_i|\rho_{\text{e-t}}|t_it_i\rangle & \sum_i\langle t_it_i|\rho_{\text{e-t}}|t_it_{i+1}\rangle & \sum_i\langle t_it_i|\rho_{\text{e-t}}|t_{i+1}t_i\rangle & \sum_i\langle t_it_i|\rho_{\text{e-t}}|t_{i+1}t_{i+1}\rangle \\ \ddots & \sum_i\langle t_it_{i+1}|\rho_{\text{e-t}}|t_it_{i+1}\rangle & \sum_i\langle t_it_{i+1}|\rho_{\text{e-t}}|t_{i+1}t_i\rangle & \sum_i\langle t_it_{i+1}|\rho_{\text{e-t}}|t_{i+1}t_{i+1}\rangle \\ \ddots & \ddots & \sum_i\langle t_{i+1}t_i|\rho_{\text{e-t}}|t_{i+1}t_i\rangle & \sum_i\langle t_{i+1}t_i|\rho_{\text{e-t}}|t_{i+1}t_{i+1}\rangle \\ c.c. & \ddots & \ddots & \sum_i\langle t_{i+1}t_{i+1}|\rho_{\text{e-t}}|t_{i+1}t_{i+1}\rangle \end{pmatrix} \tag{11}$$

In this sense, the experimental results can be understood as averages over a larger state space in the energy-time domain. Henceforth we refer to the effective density matrix with time-delayed basis states $|t\rangle$ and $|t+\tau\rangle$:

$$\rho'_{\text{e-t}} = \begin{matrix} \\ t_\mathrm{A}t_\mathrm{B} \\ t_\mathrm{A}(t_\mathrm{B}+\tau) \\ (t_\mathrm{A}+\tau)t_\mathrm{B} \\ (t_\mathrm{A}+\tau)(t_\mathrm{B}+\tau) \end{matrix} \begin{matrix} \begin{matrix} t_\mathrm{A}t_\mathrm{B} & t_\mathrm{A}(t_\mathrm{B}+\tau) & (t_\mathrm{A}+\tau)t_\mathrm{B} & (t_\mathrm{A}+\tau)(t_\mathrm{B}+\tau) \end{matrix} \\ \begin{pmatrix} \rho'_{0000} & \rho'_{0001} & \rho'_{0010} & \rho'_{0011} \\ \ddots & \rho'_{0101} & \rho'_{0110} & \rho'_{0111} \\ \ddots & \ddots & \rho'_{1010} & \rho'_{1011} \\ c.c. & \ddots & \ddots & \rho'_{1111} \end{pmatrix} \end{matrix} \tag{12}$$

In order to simplify the following discussion, we replace the time-shift operation, which would otherwise lead out of the two-dimensional energy-time subspace (i.e $\hat{\tau}|t+\tau\rangle \rightarrow |t+2\tau\rangle$) with a NOT operation which transforms the new energy-time basis states $|t\rangle$ and $|t+\tau\rangle$ as

$$
\begin{aligned}
\hat{\tau}|t\rangle &= |t+\tau\rangle \\
\hat{\tau}|t+\tau\rangle &= |t\rangle
\end{aligned} \tag{13}
$$

In doing so we neglect possible edge effects on timescales of the order of the pump coherence time. However, such effects would result in a decrease of coherence, such that they are without any consequence for our main objective of establishing lower bounds on entanglement. With this, the state of the effectively 4-dimensional system after traversing Alice's and Bob's calcite crystals can now be written as:

$$
\tilde{\rho}_{\text{pol,e-t}} = \mathcal{T}_{\text{A}}\mathcal{T}_{\text{B}}\left\{|\Phi^+\rangle\langle\Phi^+| \otimes \rho'_{\text{e-t}}\right\}\mathcal{T}_{\text{A}}\mathcal{T}_{\text{B}} \tag{14}
$$

Since the difference between adjacent time bins $\tau \sim 2\,\text{ps}$ was significantly shorter than the timing jitter of the detection system, the detection does not grant direct access to the exact emission time bins. Consequently we must perform a partial trace over the energy-time degree of freedom in order to obtain the reduced polarization density matrix ($\tilde{\rho}_{\text{pol,r}} = \text{Tr}_{\text{e-t}}(\tilde{\rho}_{\text{pol,e-t}})$) that can be accessed experimentally. The matrix can be written explicitly as:

$$
\tilde{\rho}_{\text{pol,r}} = \begin{array}{c} \\ \text{H}_\text{A}\text{H}_\text{B} \\ \text{H}_\text{A}\text{V}_\text{B} \\ \text{V}_\text{A}\text{H}_\text{B} \\ \text{V}_\text{A}\text{V}_\text{B} \end{array}
\begin{array}{c} \begin{array}{cccc} \text{H}_\text{A}\text{H}_\text{B} & \text{H}_\text{A}\text{V}_\text{B} & \text{V}_\text{A}\text{H}_\text{B} & \text{V}_\text{A}\text{V}_\text{B} \end{array} \\
\begin{pmatrix}
\frac{1}{2} & 0 & 0 & \frac{\text{Tr}(\hat{\tau}_\text{A}\hat{\tau}_\text{B}\rho'_{\text{e-t}})}{2} \\
0 & 0 & 0 & 0 \\
0 & 0 & 0 & 0 \\
\frac{\text{Tr}(\rho'_{\text{e-t}}\hat{\tau}_\text{A}\hat{\tau}_\text{B})}{2} & 0 & 0 & \frac{1}{2}
\end{pmatrix} \end{array} \tag{15}
$$

where we have used $\text{Tr}(\rho) = 1$, $\hat{\tau}^{-1} = \hat{\tau}$, and the invariance of the trace under cyclic permutation. As shown previously, the off-diagonal terms of the polarization density matrix, which determine the two-photon coherence, are now related to the off-diagonal terms of the energy-time density matrix:

$$
\text{Tr}(\rho'_{\text{e-t}}\hat{\tau}_\text{A}\hat{\tau}_\text{B}) = 2\,\text{Re}(\rho'_{0110} + \rho'_{1100}) \tag{16}
$$

In other words, polarization coherence in the reduced polarization density matrix is a direct consequence of coherence in the energy-time domain. Note that the fringe visibility in the post-selection free Franson interferometers will also be limited by the fidelity of the polarization-entangled state compared to a maximally entangled state. Using this interpretation, the visibility of the polarization correlations impose a lower bound on the visibility in the energy-time domain.

$$
V^{\phi}_{\text{e-t}} = \max_{\phi}\left[\langle\sigma^A_\phi \otimes \sigma^B_\text{x}\rangle\right] = \max_{\phi}\left[e^{i\phi} + e^{-i\phi}\right]\text{Re}(\rho'_{1100} + \rho'_{0110}) \tag{17}
$$

Criteria for 2-dimensional entanglement based on experimental visibilities

In the previous section we saw how individual visibility measurements in the polarization and energy-time subspace relate to certain elements of the respective subsystem density matrices. In the following we use these results to determine lower bounds on the concurrence $\mathcal{C}(\rho)$ and entanglement of formation $E_{\text{oF}}(\rho)$ in the polarization and energy-time-entangled subspaces. These values will then serve to establish a lower bound for the Bell-state fidelity $\mathcal{F}(\rho)$ of the entire system. In Ref. [6] easily computable lower bounds for the concurrence of mixed states that have an experimental implementation were derived. They are given by

$$\mathcal{C}(\rho) \geq \frac{2\sqrt{2}}{\sqrt{d(d-1)}} \sum_{i,j>i} \left(|\langle ii|\rho|jj\rangle| - \sqrt{\langle ij|\rho|ij\rangle\langle ji|\rho|ji\rangle} \right), \tag{18}$$

where d is the local dimension of ρ. It is possible to relax this lower bound by linearizing the above ineq. (18). Since for all $a \in \mathbb{C}$ and for all $b, c \in \mathbb{R}$ it is true that $|a| \geq \text{Re}(a)$ and $\sqrt{bc} \geq \frac{1}{2}(b+c)$, it follows that

$$\mathcal{C}(\rho) \geq \frac{2\sqrt{2}}{\sqrt{d(d-1)}} \sum_{i,j>i} \left(\text{Re}\left(\langle ii|\rho|jj\rangle\right) - \frac{1}{2}\left(\langle ij|\rho|ij\rangle + \langle ji|\rho|ji\rangle\right) \right). \tag{19}$$

By defining the operator $W(d)$ that acts on a d^2-dimensional Hilbert space as

$$W(d) := \sqrt{\frac{2}{d(d-1)}} \sum_{i,j>i} \left(2|jj\rangle\langle ii| - |ij\rangle\langle ij| - |ji\rangle\langle ji|\right), \tag{20}$$

we can rewrite ineq. (19) as

$$\mathcal{C}(\rho) \geq \text{Re}\left[\text{Tr}(\rho W(d))\right]. \tag{21}$$

We define $\mathcal{C}_{\text{lin}}(\rho)$ as the right-hand side of the above ineq. (21), which is also a lower bound for the concurrence that can be measured experimentally.

Another useful measure of entanglement is the entanglement of formation $E_{\text{oF}}(\rho)$, which represents the minimal number of maximally entangled bits (ebits) required to produce ρ via an arbitrary local operations and classical communication (LOCC) procedure. It can be shown [7] that the entanglement of formation is lower bounded by the concurrence according to:

$$E_{\text{oF}}(\rho) \geq -\log\left(1 - \frac{\mathcal{C}(\rho)^2}{2}\right), \tag{22}$$

Polarization entanglement criterion based on experimental visibility For the sake of brevity let us assume that the maximum correlation in Eq. 7 is attained for $\phi = 0$[3], so that:

$$V_{\text{pol}}^{\phi} = 2\left[\text{Re}(\rho_{0011} + \rho_{0110})\right] \tag{23}$$

[3] Alternatively, one could incorporate the phase into the definition of the concurrence or the density matrix.

In the following, we show that the linear concurrence is lower bounded by the visibilities via:

$$V_{\text{pol}}^{\phi} + V_{\text{pol}}^{\text{H/V}} - 1 \leq \mathcal{C}_{\text{lin}}(\rho_{\text{pol}}) \tag{24}$$

The experimental visibilities were $V_{\text{pol}}^{\text{H/V}} = 99.33 \pm 0.015\%$ in the linear H/V measurement basis and $V_{\text{pol}}^{\phi} = 98.5 \pm 0.15\%$ in the coherent superposition basis, respectively. We can thus infer a lower bound $\mathcal{C}_{\text{lin}}(\rho_{\text{pol}}) \geq 0.9788 \pm 0.0015$ for the concurrence. Inserting into Eq. 22 we see that this corresponds to a mininmum of $E_{\text{oF}}(\rho_{\text{pol}}) > 0.94 \pm 0.004$ ebits of entanglement of formation.

Proposition: The concurrence in polarization space is lower bounded via Eq. 24.

Proof: In the two-dimensional case the linearized concurrence (Eq. 19) reads:

$$\mathcal{C}_{\text{lin}}(\rho_{\text{pol}}) = 2\,\text{Re}(\rho_{0011}) - (\rho_{1010} + \rho_{0101}) \tag{25}$$

Inserting 25 on the r.h.s of 24 and using Eq. 6 and Eq. 23 for the visibilities, we must show that:

$$2\ \text{Re}(\rho_{0011}) + 2\ \text{Re}(\rho_{0110}) + \rho_{0000} - \rho_{0101} - \rho_{1010} + \rho_{1111} - 1 \leq 2\,\text{Re}(\rho_{0011}) - (\rho_{1010} + \rho_{0101}) \tag{26}$$

Re-ordering the terms, we obtain:

$$2\ \text{Re}(\rho_{0110}) + \rho_{0000} + \rho_{1111} \leq 1 \tag{27}$$

Now, inserting

$$\text{Tr}(\rho_{\text{pol}}) = \rho_{0000} + \rho_{1111} + \rho_{1010} + \rho_{0101} = 1 \tag{28}$$

on the r.h.s, the proof reduces to showing that:

$$\text{Re}(\rho_{0110}) \leq \frac{1}{2}(\rho_{0101} + \rho_{1010}) \tag{29}$$

The l.h.s can be upper bounded using the Cauchy-Schwartz inequality:

$$\text{Re}(\rho_{0110}) \leq |\rho_{0110}| \leq \sqrt{\rho_{1010}\rho_{0101}} \tag{30}$$

which results in:

$$\sqrt{\rho_{1010}\rho_{0101}} \leq \frac{1}{2}(\rho_{1010} + \rho_{0101}) \tag{31}$$

which is true for any two positive numbers, thus concluding the proof.

Energy-time entanglement criterion based on experimental visibility Since the adjacent time bins were too close to be resolved directly we did not measure the visibility in the computational basis. Hence we cannot use Eq. 24. Note that a lower bound on the visibility in the computational basis could be obtained via the measurement of the coincidence-to-accidental ratio (CAR) in the experiment if we assume that path-length fluctuations due to atmospheric turbulence are constant within the electronic coincidence window of 1ns. We chose to invoke a strictly weaker assumption: The matrix element ρ'_{0110} is related to coherence of photons that were emitted from the crystal with a relative delay that exceeds the coherence time (i.e. accidental coincidences). So according to the definition of the coherence time, there is no phase relationship between these pairs and we can safely assume $\rho'_{0110} \approx 0$. In other words, we assume that the free-space channel does not induce such coherence. We believe that, while this assumption precludes a certification of entanglement that meets the requirements for quantum cryptography, it is physically meaningful and completely justified in our proof of concept experiment. Under this assumption Eq. 17 reduces to:

$$V^{\phi}_{\text{e-t}} = 2\,\mathrm{Re}(\rho'_{1100}) \tag{32}$$

In the following, we show that the linear concurrence is lower bounded via:

$$2\,V^{\phi}_{\text{e-t}} - 1 \leq \mathcal{C}_{\text{lin}}(\rho_{\text{e-t}}) \tag{33}$$

Inserting the experimental Franson interference visibility of $V^{\phi}_{\text{e-t}} = 95.6 \pm 0.3\%$, we obtain $\mathcal{C}_{\text{lin}}(\rho_{\text{e-t}}) \geq 0.912 \pm 0.006$ and $E_{\text{oF}}(\rho_{\text{e-t}}) > 0.776 \pm 0.014$ ebits of entanglement of formation.

Proposition: The concurrence in the energy-time subspace is lower bounded via Eq. 33.

Proof: Inserting Eq. 32 on the l.h.s and Eq. 25 on the r.h.s of Eq. 33, we must show that:

$$4\,\mathrm{Re}(\rho'_{1100}) - 1 \leq 2\,\mathrm{Re}(\rho'_{1100}) - (\rho'_{1010} + \rho'_{0101}) \tag{34}$$

re-ordering we can write this as:

$$2\,\mathrm{Re}(\rho'_{1100}) + (\rho'_{1010} + \rho'_{0101}) \leq 1 \tag{35}$$

Inserting

$$\mathrm{Tr}(\rho_{\text{e-t}}) = \rho'_{0000} + \rho'_{1111} + \rho'_{1010} + \rho'_{0101} = 1 \tag{36}$$

on the r.h.s, the proof reduces to showing that:

$$\mathrm{Re}(\rho'_{1100}) \leq \frac{\rho'_{1111} + \rho'_{0000}}{2} \tag{37}$$

The l.h.s can be upper bounded using the Cauchy-Schwartz inequality:

$$\mathrm{Re}(\rho'_{1100}) \leq |\rho'_{1100}| \leq \sqrt{\rho'_{1111}\rho'_{0000}} \tag{38}$$

which results in:

$$\sqrt{\rho'_{1111}\rho'_{0000}} \leq \frac{1}{2}(\rho'_{1111} + \rho'_{0000}) \tag{39}$$

which is true for any two positive numbers, thus concluding the proof.

Entanglement criterion for combined state space based on entanglement in subspaces

In the previous section we used the results of individual visibility measurements in the polarization and energy-time subspace to determine a lower bound on the entanglement of formation $E_{\mathrm{oF}}(\rho)$, concurrence $\mathcal{C}(\rho)$, and Bell-state fidelity $\mathcal{F}(\rho)$ in each subspace. In the following section we show that the results of these individual characterizations can be used to establish a lower bound on the entanglement of the entire hyperentangled system, and thus certify genuine high-dimensional entanglement.

The concurrence of a pure bipartite state $|\psi\rangle$ acting on the finite-dimensional Hilbert space $\mathcal{H}_{\mathrm{A}} \otimes \mathcal{H}_{\mathrm{B}}$ is a measure of entanglement defined as [8]

$$\mathcal{C}(|\psi\rangle) = \sqrt{2(1 - \mathrm{Tr}(\rho_{\mathrm{A}}^2))}, \tag{40}$$

where ρ_{A} is the reduced state over the subspace $\mathcal{H}_{\mathrm{B}}$. Its generalization for bipartite mixed states $\rho = \sum_i p_i |\psi_i\rangle\langle\psi_i|$ follows from the convex roof construction,

$$\mathcal{C}(\rho) = \inf_{\{p_i, |\psi_i\rangle\}} \sum_i p_i \mathcal{C}(|\psi_i\rangle), \tag{41}$$

where the infimum is obtained over all possible decompositions of ρ. Given two bipartite mixed states, ρ and σ, it follows from the definition of the concurrence the subadditivity relation

$$\mathcal{C}(\rho \otimes \sigma) \leq \mathcal{C}(\rho) + \mathcal{C}(\sigma). \tag{42}$$

This quantity is in general very hard to compute, however, with Eq. 21 we have an easily computable lower bound $\mathcal{C}_{\mathrm{lin}}(\rho)$ for the concurrence.

Let $\rho_{\mathrm{A}} \in \mathcal{L}(\mathcal{H}^{d_{\mathrm{A}}} \otimes \mathcal{H}^{d_{\mathrm{A}}})$ and $\rho_{\mathrm{B}} \in \mathcal{L}(\mathcal{H}^{d_{\mathrm{B}}} \otimes \mathcal{H}^{d_{\mathrm{B}}})$, be two unknown states whose values for $\mathcal{C}_{\mathrm{lin}}(\rho_{\mathrm{A}})$ and $\mathcal{C}_{\mathrm{lin}}(\rho_{\mathrm{B}})$ have been measured. Let $\rho_{\mathrm{AB}} \in \mathcal{L}(\mathcal{H}^{d_{\mathrm{A}}d_{\mathrm{B}}} \otimes \mathcal{H}^{d_{\mathrm{A}}d_{\mathrm{B}}})$ be also an unknown state whose reduced states are $\rho_{\mathrm{A}} = \mathrm{Tr}_{\mathrm{B}}(\rho_{\mathrm{AB}})$ and $\rho_{\mathrm{B}} = \mathrm{Tr}_{\mathrm{A}}(\rho_{\mathrm{AB}})$, and whose concurrence $\mathcal{C}(\rho_{\mathrm{AB}})$ one is interested in calculating. Because of the subadditivy character of the concurrence, it is not possible to simply add the known values of $\mathcal{C}_{\mathrm{lin}}(\rho_{\mathrm{A}})$ and $\mathcal{C}_{\mathrm{lin}}(\rho_{\mathrm{B}})$ in order to estimate $\mathcal{C}(\rho_{\mathrm{AB}})$; it is necessary to calculate a lower bound for this quantity. We now show how to derive a useful lower bound for the concurrence $\mathcal{C}(\rho_{\mathrm{AB}})$ given the experimentally accessible values $\mathcal{C}_{\mathrm{lin}}(\rho_{\mathrm{A}})$ and $\mathcal{C}_{\mathrm{lin}}(\rho_{\mathrm{B}})$.

First, notice that the reduced states ρ_{A} and ρ_{B} must satisfy

$$\mathcal{C}_{\mathrm{lin}}(\rho_{\mathrm{A}}) = \mathrm{Re}\left[\mathrm{Tr}(\rho_{\mathrm{A}} W(d_{\mathrm{A}}))\right] \tag{43}$$

and

$$\mathcal{C}_{\mathrm{lin}}(\rho_{\mathrm{B}}) = \mathrm{Re}\left[\mathrm{Tr}(\rho_{\mathrm{B}} W(d_{\mathrm{B}}))\right]. \tag{44}$$

Since $\mathcal{C}(\rho_{\mathrm{AB}}) \geq \mathcal{C}_{\mathrm{lin}}(\rho_{\mathrm{AB}})$, we calculate a lower bound for $\mathcal{C}(\rho_{\mathrm{AB}})$ by minimizing $\mathcal{C}_{\mathrm{lin}}(\rho_{\mathrm{AB}})$ over all possible states ρ_{AB} whose reduced states ρ_{A} and ρ_{B} satisfy conditions (43) and (44). Namely,

$$\mathcal{C}(\rho_{\mathrm{AB}}) \geq \min_{\rho_{\mathrm{AB}}} \mathrm{Re}\left[\mathrm{Tr}(\rho_{\mathrm{AB}} W(d_{\mathrm{A}} d_{\mathrm{B}}))\right]. \tag{45}$$

This minimization problem can now be solved by semi-definite programming (SDP). Defining C_{lb} as the right-hand side of the above ineq. (45), we write the following SDP:

$$\begin{aligned}
\text{given} \quad & \mathcal{C}_{\mathrm{lin}}(\rho_{\mathrm{A}}), \mathcal{C}_{\mathrm{lin}}(\rho_{\mathrm{B}}) \\
C_{\mathrm{lb}} = \min_{\rho_{\mathrm{AB}}} \quad & \mathrm{Re}\left[\mathrm{Tr}(\rho_{\mathrm{AB}} W(d_{\mathrm{A}} d_{\mathrm{B}}))\right] \\
\text{s.t.} \quad & \rho_{\mathrm{AB}} \geq 0,\ \mathrm{Tr}(\rho_{\mathrm{AB}}) = 1, \\
& \mathcal{C}_{\mathrm{lin}}(\rho_{\mathrm{A}}) = \mathrm{Re}\left[\mathrm{Tr}(\mathrm{Tr}_{\mathrm{B}}(\rho_{\mathrm{AB}}) W(d_{\mathrm{A}}))\right], \\
& \mathcal{C}_{\mathrm{lin}}(\rho_{\mathrm{B}}) = \mathrm{Re}\left[\mathrm{Tr}(\mathrm{Tr}_{\mathrm{A}}(\rho_{\mathrm{AB}}) W(d_{\mathrm{B}}))\right].
\end{aligned} \tag{46}$$

The solution C_{lb} of this SDP is a lower bound for $\mathcal{C}(\rho_{\mathrm{AB}})$. Since the entanglement of formation $E_{\mathrm{oF}}(\rho)$ is lower bounded by the concurrence according to

$$E_{\mathrm{oF}}(\rho) \geq -\log\left(1 - \frac{\mathcal{C}(\rho)^2}{2}\right), \tag{47}$$

the lower bound C_{lb} for the concurrence $\mathcal{C}(\rho_{\mathrm{AB}})$ allows us to also calculate a lower bound for the entanglement of formation $E_{\mathrm{oF}}(\rho_{\mathrm{AB}})$.

The fidelity to the d-dimensional maximally entangled state $|\Phi_d^+\rangle = \frac{1}{\sqrt{d}} \sum_i |ii\rangle$ can also be lower bounded from the subspace concurrences by altering the target function of the above SDP. Let F_{lb} be

$$F_{\mathrm{lb}} = \min_{\rho_{\mathrm{AB}}} \mathrm{Tr}(\rho_{\mathrm{AB}} |\Phi_d^+\rangle\langle\Phi_d^+|), \tag{48}$$

where the minimum is taken over all states ρ_{AB} that satisfy conditions (43) and (44) for the subspace concurrences. Then, we can write the following SDP:

$$\begin{aligned}
\text{given} \quad & \mathcal{C}_{\mathrm{lin}}(\rho_{\mathrm{A}}), \mathcal{C}_{\mathrm{lin}}(\rho_{\mathrm{B}}) \\
F_{\mathrm{lb}} = \min_{\rho_{\mathrm{AB}}} \quad & \mathrm{Tr}(\rho_{\mathrm{AB}} |\Phi_d^+\rangle\langle\Phi_d^+|) \\
\text{s.t.} \quad & \rho_{\mathrm{AB}} \geq 0,\ \mathrm{Tr}(\rho_{\mathrm{AB}}) = 1, \\
& \mathcal{C}_{\mathrm{lin}}(\rho_{\mathrm{A}}) = \mathrm{Re}\left[\mathrm{Tr}(\mathrm{Tr}_{\mathrm{B}}(\rho_{\mathrm{AB}}) W(d_{\mathrm{A}}))\right], \\
& \mathcal{C}_{\mathrm{lin}}(\rho_{\mathrm{B}}) = \mathrm{Re}\left[\mathrm{Tr}(\mathrm{Tr}_{\mathrm{A}}(\rho_{\mathrm{AB}}) W(d_{\mathrm{B}}))\right].
\end{aligned} \tag{49}$$

The solution F_{lb} is a lower bound for the fidelity to the maximally entangled state $\mathcal{F}(\rho_{\text{AB}}) \geq F_{\text{lb}}$.

For ρ_{pol} and $\rho_{\text{e-t}}$ being entangled states in the polarization and time-energy degrees of freedom, respectively, we have showed how to calculate $\mathcal{C}_{\text{lin}}(\rho_{\text{pol}})$ and $\mathcal{C}_{\text{lin}}(\rho_{\text{e-t}})$ from the fringe visibilities V_{pol} and $V_{\text{e-t}}$. For values of $\mathcal{C}_{\text{lin}}(\rho_{\text{pol}}) = 0.977$ and $\mathcal{C}_{\text{lin}}(\rho_{\text{e-t}}) = 0.906$, we obtain the lower bound $\mathcal{C}(\rho) \geq 1.1299$ for the concurrence and $E_{\text{oF}}(\rho) \geq 1.4671$ for the entanglement of formation of the hyperentangled state ρ. This bound is sufficient to guarantee $d = 3$ entanglement [9]. For the same values of $\mathcal{C}_{\text{lin}}(\rho_{\text{pol}})$ and $\mathcal{C}_{\text{lin}}(\rho_{\text{e-t}})$ we obtain a lower bound for the fidelity to the maximally entangled state in $d = 4$ of $\mathcal{F}(\rho) \geq 0.9419$, which is enough to certify $d = 4$ entanglement [10], an even higher dimensionality.

For these inputs values of $\mathcal{C}_{\text{lin}}(\rho_{\text{pol}})$ and $\mathcal{C}_{\text{lin}}(\rho_{\text{e-t}})$, and $d_{\text{A}} = d_{\text{B}} = 2$, there exists a strictly feasible point ρ^* that returns $\text{Re}\left[\text{Tr}(\rho^* W(4))\right] = 1.14$ and $\text{Tr}(\rho^* |\Phi_4^+\rangle\langle\Phi_4^+|) = 0.95$ which guarantees that SDPs (46) and (49) satisfy the condition of strong duality [11]. This is proof that the optimal values $C_{\text{lb}} = 1.1284$ and $F_{\text{lb}} = 0.9410$ were indeed achieved by both primal and dual problems. Since these are finite values, it is guaranteed that the true minimum of both optimization problems was attained.

Supplementary Figures

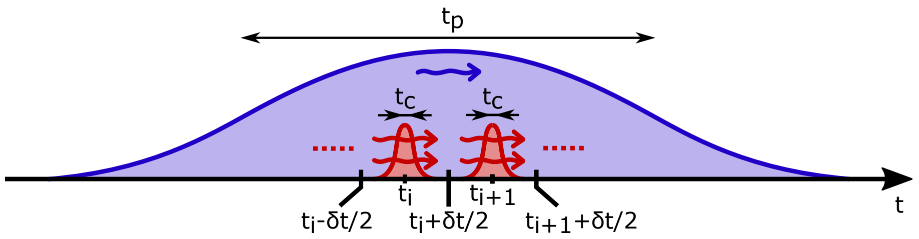

Supplementary Figure 1: Illustration of relevant coherence times in energy-time space. The pump photon is indicated by a blue envelope with coherence time t_{p}, while two possible SPDC photon pair emission times are indicated by red envelopes with coherence times $t_{\mathrm{c}} \ll t_{\mathrm{p}}$. Note, that the coherence times are not drawn to scale. For our analysis of the energy-time entangled state, the emission time of the photon pairs are grouped into non-overlapping time bins of length δt.

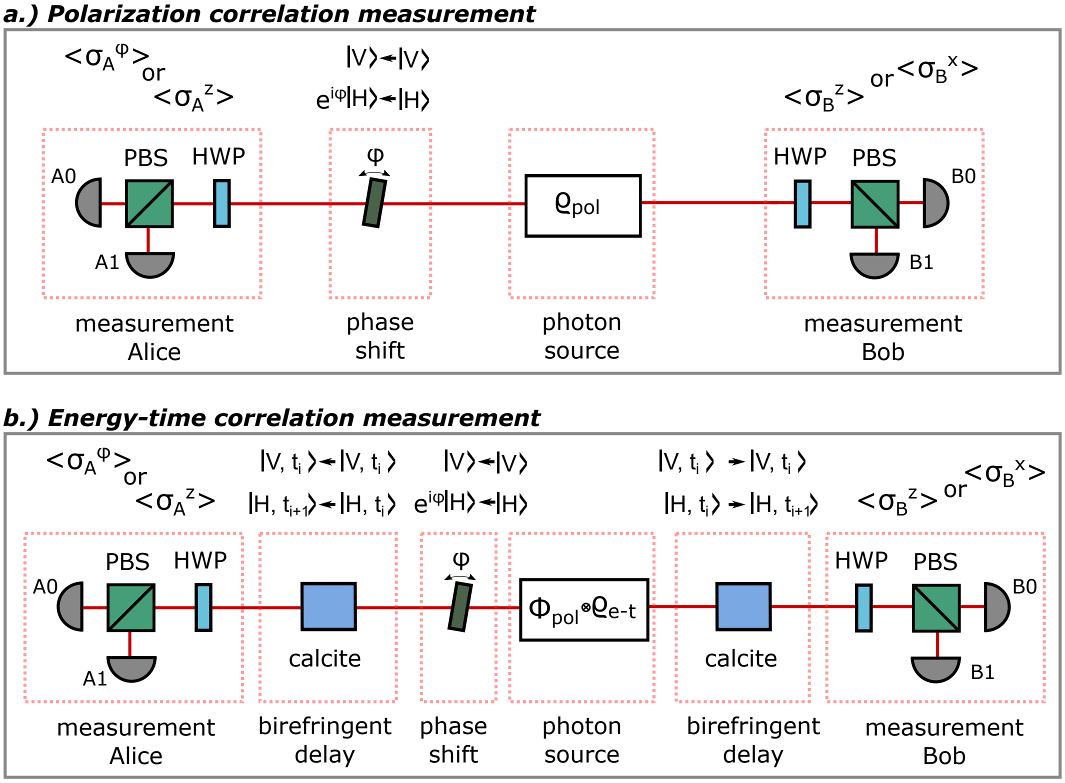

Supplementary Figure 2: Experimental setup for polarization and energy-time correlation measurements. Hyperentangled photons are distributed to Alice and Bob. Alice applies an additional phase shift ϕ by tilting a birefringent crystal. Alice and Bob evaluate the visibility of the two-photon correlation functions in the computational basis $V_{\text{pol}}^{\text{H/V}}=\langle\sigma_{\text{A}}^{\text{z}}\otimes\sigma_{\text{B}}^{\text{z}}\rangle$ and the superposition basis $V_{\text{pol}}^{\phi}=\max_{\phi}[\langle\sigma_{\text{A}}^{\phi}\otimes\sigma_{\text{B}}^{\text{x}}\rangle]$. a.) Polarization correlation measurements b.) Energy-time correlation measurement: In order to assess the energy-time coherence an additional calcite crystal is added in Alice's and Bob's measurement setup. The calcite crystal introduces a polarization-dependent delay that couples the polarization and energy-time DOF, i.e. maps coherence in the energy-time state to coherence in the polarization DOF.

Supplementary References

1. Shih, Y. Entangled biphoton source - property and preparation. *Reports on Progress in Physics* **66**, 1009 (2003).

2. Strekalov, D., Pittman, T., Sergienko, A., Shih, Y. & Kwiat, P. Postselection-free energy-time entanglement. *Physical Review A* **54**, R1 (1996).

3. Franson, J. D. Bell inequality for position and time. *Physical Review Letters* **62**, 2205–2208 (1989).

4. Xie, Z. *et al.* Harnessing high-dimensional hyperentanglement through a biphoton frequency comb. *Nature Photonics* **9**, 536–542 (2015).

5. Martin, A. *et al.* Quantifying photonic high-dimensional entanglement. *Phys. Rev. Lett.* **118**, 110501 (2017).

6. Ma, Z.-H. *et al.* Measure of genuine multipartite entanglement with computable lower bounds. *Phys. Rev. A* **83**, 062325 (2011).

7. Wootters, W. K. Entanglement of formation and concurrence. *Quantum Information & Computation* **1**, 27–44 (2001).

8. Mintert, F., Kuś, M. & Buchleitner, A. Concurrence of mixed multipartite quantum states. *Phys. Rev. Lett.* **95**, 260502 (2005).

9. Huber, M. & de Vicente, J. I. Structure of multidimensional entanglement in multipartite systems. *Phys. Rev. Lett.* **110**, 030501 (2013).

10. Fickler, R. *et al.* Interface between path and orbital angular momentum entanglement for high-dimensional photonic quantum information. *Nature Communications* **5**, 4502 (2014).

11. Boyd, S. & Vandenberghe, L. *Convex Optimization* (Cambridge University Press, New York, NY, USA, 2004).
